# Restless nights when sick: ectoparasite infections alter rest–activity cycles of diurnal fish hosts

**DOI:** 10.1017/S0031182023001324

**Published:** 2024-03

**Authors:** Elissavet A. Arapi, Michael Reynolds, Amy R. Ellison, Jo Cable

**Affiliations:** 1 School of Biosciences, Cardiff University, Cardiff CF10 3AX, UK; 2 School of Natural Sciences, Bangor University, Bangor LL57 2DG, UK

**Keywords:** circadian rhythms, disease susceptibility, host health, infection, nocturnal activity, rest deprivation

## Abstract

Circadian rhythms are timekeeping mechanisms responsible for an array of biological processes. Disruption of such cycles can detrimentally affect animal health. Circadian rhythms are critical in the co-evolution of host–parasite systems, as synchronization of parasite rhythms to the host can influence infection dynamics and transmission potential. This study examines the circadian rhythms in behaviour and activity of a model fish species (*Poecilia reticulata*) in isolation and in shoals, both when uninfected and infected with an ectoparasite (*Gyrodactylus turnbulli*). Additionally, the rhythmical variance of parasite activity under different light conditions as well as rhythmical variance in parasite transmissibility was explored. Overall, infection alters the circadian rhythm of fish, causing nocturnal restlessness. Increased activity of gyrodactylids on the host's skin at night could potentially contribute to this elevated host activity. Whilst migration of gyrodactylids across the host's skin may have caused irritation to the host resulting in nocturnal restlessness, the disruption in guppy activity rhythm caused by the expression of host innate immunity cannot be excluded. We discuss the wider repercussions such behavioural responses to infection have for host health, the implications for animal behaviour studies of diurnal species as well as the application of chronotherapeutic approaches to aquaculture.

## Graphical abstract

**Figure:**
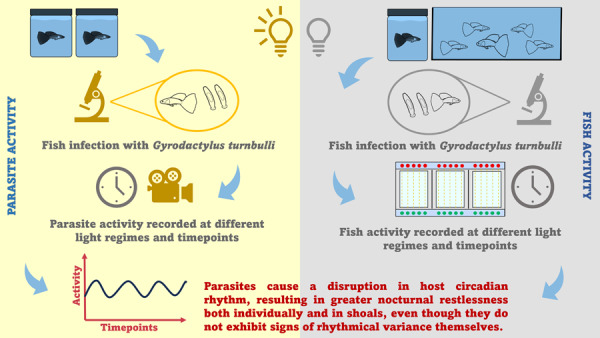

## Introduction

Circadian rhythms are intrinsic timekeeping mechanisms responsible for the cyclic repetition of metabolic, behavioural and psychological processes in all living organisms, typically over a 24-h period (Liang *et al*., 2015; Sollars and Pickard, 2015). They are endogenously generated by self-sustaining biological clocks, encoded by ‘clock genes’, and entrained by environmental cues such as light and temperature (Piggins, 2002). Their disruption can affect an array of biological activities such as rest–activity cycles, immunity and disease susceptibility (Bass and Lazar, 2016), as shown in humans if natural circadian cues are ignored due to shift work, jet-lag and general sleep deprivation (Takahashi *et al*., 2008).

Sleep is a complex enigma that serves multiple functions (Krueger *et al*., 2016), most notably provisioning critical restorative and repair processes (Adam, 1980; Benington and Craig Heller, 1995; Cirelli and Tononi, 2008; Helvig *et al*., 2016). The general assumption that all species ‘sleep’ is controversial, with some animals entering a restful state that does not necessarily fulfil descriptors depicting true sleep (Siegel, 2008). Recent evidence of true sleep (including Rapid Eye Movement sleep phase; REM), however, has now been reported in zebrafish (Leung *et al*., 2019). Furthermore, a consistent observation across taxa is that disturbances to ‘rest–activity’ cycles, and thus disruption of circadian rhythms, can have detrimental consequences for health with respect to disease, even reducing life expectancy (Kripke *et al*., 2002; Davidson, 2006).

In fish, circadian rhythms govern biological activities ranging from reproduction, spawning, smoltification and maturation to immune responses. Circadian rhythms have been observed in activity patterns of various fish of economic importance including the golden shiner (*Notemigonus crysoleucas*), goldfish (*Carassius auratus*), lake chub (*Couesius plumbeus*), Atlantic salmon (*Salmo salar*) and rainbow trout (*Oncorhynchus mykiss*) (see Reebs, 2002). In aquaculture, manipulating photoperiods, such as extending the light period in diurnal species, can improve rearing quality and promote increased growth rates (Boeuf and Le Bail, 1999). In the extreme, constant light is used to improve feed utilization (Boeuf and Le Bail, 1999) or control maturation and puberty (Taranger *et al*., 2010). However, this may have negative implications for health and disease resistance, as immune functions are often highly rhythmic, enabling organisms to mount their most efficient response at times when risk of infection or injury is highest (Ellison *et al*., 2021). Conversely, immune factors and infections can affect expression of molecular clocks (Castanon-Cervantes *et al*., 2010; Adams *et al*., 2013). So, disruption of normal circadian cycles can impact immune responses and may increase disease risks (Ellison *et al*., 2021). Given the increased understanding of the intricate link between fish body clocks and their immune systems, harnessing knowledge of circadian rhythms may be hugely beneficial against infectious diseases. However, for chronobiological approaches to tackle infectious diseases, rhythms of both the fish and their associated parasites must be considered.

Parasites can directly impact host rest–activity cycles (Ibarra-Coronado *et al*., 2015), which are associated with activation of immune defences (Preston *et al*., 2009). Moreover, individuals are most at risk of acquiring parasitic infections when sleep deprived (Bryant *et al*., 2004; Majde and Krueger, 2005). Thus, the reciprocal interaction between rest–activity cycles and immune functioning is complex (Opp, 2009). The underlying mechanism appears to be stress-related, which in turn affects the immune system, causing increased susceptibility to infection and subsequently higher mortality rates (Penev *et al*., 1998; Davidson, 2006). In fish, immune responses to infectious diseases have been extensively studied in the past. Now, increasingly more studies investigate the disruption of fish circadian rhythms by parasites, as in the case of zebrafish (*Danio rerio*), where established *Pseudoloma neurophilia* infections induced major transcriptional changes in the host brain (Midttun *et al*., 2020). However, little is known about how parasites might alter fish resting periods and the long-term implications of disrupted circadian rhythms.

Parasite circadian rhythms are critical in the co-evolution of host–parasite systems, as synchronization of their rhythms can influence infection dynamics and transmission potential (O'Donnell *et al*., 2011). Parasite circadian rhythms are apparent in oviposition (*Schistosoma haematobium* see Theron and Combes, 1995; *Passalarus ambiguous* see Rinaldi *et al*., 2007), timing of asexual reproduction (*Plasmodium chabaudi* see Mideo *et al*., 2013) as well as expression of certain metabolism genes (*Trypanosoma brucei* see Rijo-Ferreira *et al*., 2017). Circadian rhythms have also been implicated in detachment of parasites from their host (Doube, 1975), as well as host immune evasion by secretion of chemical signals (DuRant *et al*., 2015). For monogenean ectoparasites, rhythmical variance has been observed in egg laying and hatching (*Discocotyle sagittata* see Gannicott and Tinsley, 1997*; Entobdella soleae* see Kearn, 1967; *Benedenia ludjani* see Ernst and Whittington, 1996). With circadian rhythms seemingly affecting various aspects of a parasite's life cycle, the impact of circadian rhythms on infection potential and dynamics needs to be further explored.

One of the most ubiquitous groups of monogenean parasites infecting teleost fish are the gyrodactylids. These parasites are known to infect multiple fish of aquacultural importance, including cyprinids (Zietara and Lumme, 2002) and salmonids (Harris *et al*., 2004), and can have a major economic impact on the industry. Little is known regarding daily activity rhythms of gyrodactylids, such as movement on the host and host-seeking behaviour, with the exception of 1 study which reported greater variation of *in vitro* parasite activity in dark compared to light conditions (Brooker *et al*., 2011). From a host perspective, sticklebacks (*Gasterosteus aculeatus*) were more susceptible to *Gyrodactylus gasterostei* when exposed to prolonged photoperiods; due to changes in host physiology, condition and immune responses (Whiting *et al*., 2020). However, whether gyrodactylids exhibit a light-dependant behaviour or parasite activity has true circadian rhythmicity has yet to be studied.

The current study investigates (a) the impact of an ectoparasitic infection on host rest–activity cycles, and (b) the rhythmical variance in parasite activity and behaviour. For this study, we used the tropical Trinidadian guppy (*Poecilia reticulata*)–*Gyrodactylus turnbulli* model; a system that has been subject to extensive epidemiological and behavioural investigations (Bakke *et al*., 2007). Although this parasite has been known to cause behavioural modifications in its typically diurnal host (e.g. foraging and swimming performance; Cable *et al*., 2002; Kolluru *et al*., 2009), the daily dynamics of guppy-gyrodactylid interactions have, until now, been overlooked. Therefore, we are exploring the behaviour of infected hosts compared to their uninfected conspecifics both when isolated and in shoals and we discuss the implications that this may have for host health and aquaculture in general.

## Materials and methods

### Host and parasite origins and maintenance

Trinidadian guppies (*P. reticulata*) originating from the Lower Aripo River, Trinidad (wild-type strain), or from a commercial wholesaler (ornamental strain) were transported to Cardiff University Aquarium. Fish stocks were housed separate in 70 L tanks of dechlorinated water (approx. 1 fish/1 L water, as recommended by OATA; Ornamental Aquatic Trade Association), in 24 ± 1°C in a 12:12 h light: dark regime and fed daily with Aquarian® tropical fish flakes supplemented with live *Daphnia magna* and freshly hatched *Artemia* nauplii. For all experiments, female or juvenile guppies were used and size-matched to avoid size variability, which is known to affect parasite load (Cable and van Oosterhout, 2007). For each experiment, only 1 fish stock and single sex fish were used, to avoid confounding variables.

For experimental infections, the *Gt3* strain of *G. turnbulli* was used; isolated in 1997 from, and subsequently maintained on ornamental guppies (as in Stewart *et al*., 2017). For all experimental infections, a sacrificed donor was placed close to a recipient fish anaesthetized with 0.2% tricaine methanesulfonate (MS222). Direct contact between hosts facilitated transfer of gyrodactylids, as observed under a dissecting microscope with fibre optic illumination. Fish were infected with 30 parasites each, representative of burdens reached after 5 days for an individually isolated fish experimentally infected with 2 worms on Day 0 (e.g. Van Oosterhout *et al*., 2003).

### Experimental design

Overall, 4 experiments were performed: 2 compared the rhythmical activity of the guppy host when uninfected and infected with *G. turnbulli* and 2 explored the rhythmical variance in activity of the actual parasite. For all experiments, we report the Zeitgeber Time (ZT) system, where ZT is a unit of time based on light Zeitgeber. The ZT denotes when the lights go on and off, in this case, ZT0 denotes lights on and ZT12 lights off (Karatsoreos and Silver, 2017). There was no light fade to simulate sunrise/sunset conditions. For nocturnal observations, infrared lights (light intensity 1.2–1.3 Lux; Precision Gold Digital Light Meter) were used compared to the white light (500 Lux intensity) used during the day, as infrared illumination is invisible to the animals being observed but visible to infrared cameras (Widder *et al*., 2005).

For all experiments, uninfected control fish were sham infected to account for handling time and then returned to 1 L dechlorinated water pots to recover. No anaesthetic associated mortalities occurred during this study and the anaesthesia process, with a 0.02% MS222 dose, seemed to have no effect on host and parasite behaviour and survival (Chambel *et al*., 2015). Following infection (and sham infection), fish were transferred to the experimental tank in a small glass dish containing dechlorinated water, ensuring the fish was never out of water nor was there any risk of nets dislodging the ectoparasites. Once all experimental trials concluded, infected fish were treated with an anti-helminthic drug, 0.1% Levamisole, to eliminate any parasites and then screened clear under the microscope 3 consecutive times to ensure that they were parasite-free (Schelkle *et al*., 2009).

### Automated monitoring of host behaviour

**Figure 1.**
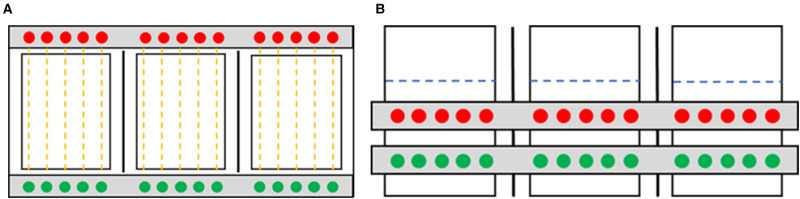
2D schematic showing the set-up of the behavioural arrays for experiment 1. (a). Birds eye view of the behavioural arrays with 2 rows of 5 infrared beams (yellow dotted line) going through each fish tank from the light emitters (green) to the light receivers (red). (b). Side view of the behavioural arrays with 2 rows of monitors outside of each tank with the light emitters going through the tank to the receivers on the other side, with light emitters and receivers alternating between rows. The water level is indicated (blue dotted line) along with the paper dividers between the tanks (black line).

Behavioural arrays used in experiment 1 for monitoring infected and uninfected individual fish consisted of 3 acrylic tanks (22 cm length × 10 cm width × 20 cm depth; Fig. 1), positioned within 2 rows of TriKinetics behavioural monitors. Each behavioural array tank was filled with 1.25 L of dechlorinated water and white card paper on each side of the tanks avoided any external disturbances to the fish. Ten infrared beams passed through each tank, 5 from the top monitor and 5 from the bottom monitor, from the infrared emitters to the receivers. The monitors were connected to the TriKinetics software, which automatically recorded how many times a fish passed through a beam within a certain time period.

### Experiment 1: impact of infection on daily activity of isolated guppies

To observe whether there is a difference in activity between uninfected and infected isolated wild-type guppies under a 12:12 h light: dark regime, female adult guppies were size-matched (15.68 ± 0.95 mm) and 2 experimental groups were created: uninfected controls (*n* = 11) and infected experimental fish (*n* = 10). Fish remained in individual 1 L containers for 7 days prior to start of the experiment. On Day 1, experimental guppies were infected with exactly 30 gyrodactylids, whilst control fish were sham infected to control for handling time. Each fish was then placed into a 1 L dechlorinated pot to recover, before being transferred to a behavioural array tank for acclimation. At 07:00, the following day (Day 2), the arrays started monitoring guppy activity every minute for 48 h. On Day 4, fish were removed from the tanks, anaesthetized and screen under the microscope. The experimental fish were screened to record their final parasite load (mean intensity 73, range 49–93) and the control fish were screened in order to ensure that no contamination had occurred, with control fish indeed remaining parasite free. Fish activity was recorded as the counts of infrared beam breaks per tank, as retrieved from the TriKinetics software and investigated hourly from Day 1 (08:00). As the arrays monitored guppy activity every minute, recordings were then averaged per hour, to follow the ZT system.

### Experiment 2: impact of infection on daily activity of guppy shoals

To observe whether there is a difference in activity between uninfected and infected wild-type guppy shoals under a 12:12 h light: dark regime, female adult guppies were size matched (13.21 ± 0.67 mm) into shoals of 5 individuals (*n* = 16 groups). Each shoal was housed in 6 L familiarization tanks for a minimum of 12 days (Griffiths and Magurran, 1997) prior to trials. On Day 1 of the experiment, each familiarized shoal was transferred to a test arena (150 cm length × 20 cm width × 16 cm depth) for a 24 h acclimation period. At 08:00 the following day (Day 2), fish were removed from the arena, and 1 guppy was anaesthetized and infected with 30 gyrodactylids, whilst the remaining 4 fish in each shoal were sham infected to account for handling time. Fish were placed in individual 1 L pots for 30 min recovery time, whilst remaining in visual contact to one another. On Day 3, an observer (partially hidden by a screen) recorded the proportion of time (sec) an infected and a randomly selected uninfected fish spent actively swimming during a 5-min focal follow over 5 time points; 3 diurnal (ZT1: 08:00, ZT6: 13:00 and ZT11: 18:00 h) and 2 nocturnal (ZT15: 22:00 and ZT18: 01:00 h). Fish were deemed actively swimming when propelling themselves forward. After the 5-min focal follow, both fish were screened to account for any parasites transfer. Data collected from the uninfected individuals were used as a control.

### Experiment 3: impact of photoperiod on parasite daily activity

To identify whether there is rhythmical variance in parasite activity under the 2 light regimes (12:12 h light: dark and 24 h constant darkness; constant darkness often used a ‘free-running’ condition – a test of endogenous circadian rhythms; Brown *et al*., 2011), we monitored the host-seeking motion of the parasite (number of probes), which is part of their exploratory behaviour (Bakke *et al*., 2007). For both light conditions (12:12 h light: dark and 24 h darkness), wild-type juvenile guppies (*n* = 60 for each experiment) were size-matched (10.75 ± 0.40/11.10 ± 0.9 mm) and each fish infected with 2 gyrodactylids, before being placed individually in 1 L dechlorinated water pots. After an acclimation period of 7 days, during which parasite number on each host increased naturally, in a 12:12 h light: dark regime, fish were anaesthetized and parasite activity recorded for a 2-min period under a dissecting microscope, using a Longse standard box camera. The activity of 3 randomly selected parasites on the fins of each fish was analysed. For the first condition, parasite activity was recorded both in light and dark depending on the ZT point, whereas for the second condition at ZT0 the light remained off, so all recordings took place in constant darkness with infrared light. Once recordings concluded, the host parasite load was also recorded. For these observations, timepoints monitored were ZT0 (07:00 h), ZT4 (11:00 h), ZT8 (15:00 h), ZT12 (19:00 h), ZT16 (23:00 h) and ZT20 (03:00 h).

### Experiment 4: impact of photoperiod on parasite transmissibility

To examine whether daily variation in parasite activity affected their transmissibility to a new host, ornamental female adult guppies (*n* = 120) were size-matched (12.94 ± 1.3 mm) into dyads. One guppy from each dyad (*n* = 60) was infected with 2 gyrodactylids and all guppies were placed individually in 1 L pots. After an acclimation period of 7 days in a 12:12 h light: dark regime, infected individuals were screened to determine their parasite load. Then, both infected and uninfected guppies from each dyad were placed together in 25 mL of dechlorinated water for 1 h, resulting in 10 dyads at each of the following time points: ZT0 (07:00 h), ZT4 (11:00 h), ZT8 (15:00 h), ZT12 (19:00 h), ZT16 (23:00 h) and ZT20 (03:00 h). After 1 h, fish were separated, anaesthetized and screened to record how many parasites had transferred from the donor to the recipient or how many parasites had been dislodged.

### Statistical analysis

**Figure 2.**
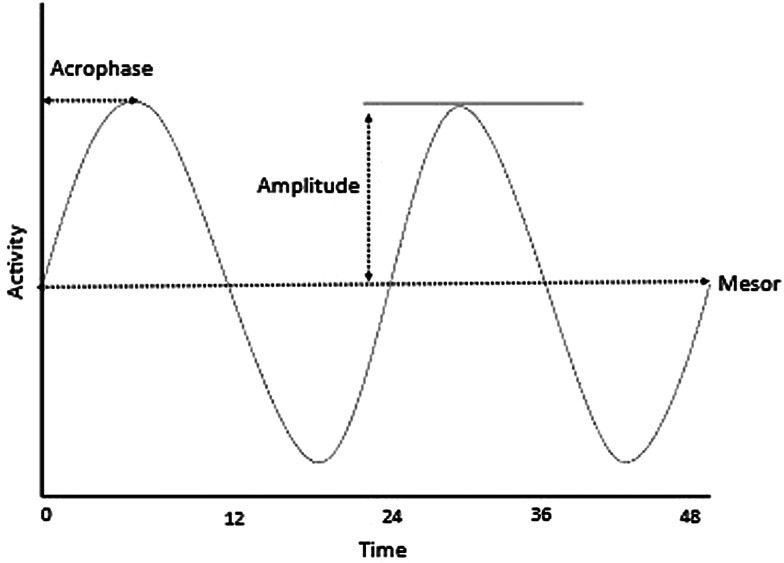
Variables assessed by the ‘circacompare’ package in each rhythm and then compared between rhythms (include Mesor, the rhythm-adjusted mean level; amplitude, half the extend of predictable variation; and acrophase the time the response variable peaks).

All statistical analyses were conducted using the R statistical software (version 4.1.1, R Core Team, 2019). To analyse the data, the following packages were used: ‘lme4’ to run Generalized Linear Mixed Models (GLMMs) (Bates *et al*., 2015), ‘emmeans’ for *post hoc* analyses (Searle *et al*., 1980), ‘ggplot2’ to visualize data (Wickham, 2009) and ‘circacompare’ to compare rhythms (Parsons *et al*., 2020). The ‘circacompare’ package was used to compare rhythms between different conditions by assessing MESOR, amplitude and acrophase across rhythms. MESOR (Midline Estimating Statistic of Rhythm) refers to the rhythm-adjusted mean level of a response variable around which a wave function oscillates, so the mean activity level over a certain period of time. Amplitude is a measure of half the extent of predictable variation within a cycle, so the activity variation from the MESOR, which is the mean, to the peak of activity. Acrophase refers to the time at which the response variable peaks; the time that it takes to go from MESOR to Amplitude (Otsuka *et al*., 2016; Parsons *et al*., 2020; Fig. 2).

For experiment 1, a GLMM fitted with ‘binomial error’ family and ‘logit’ link function assessed activity (count of infrared beam breaks) of infected and uninfected isolated guppies in light and dark conditions. Guppy activity was the dependent term in the model, and fixed effects were infection status (infected or uninfected) and light condition (light or dark). Fish number was included as a random term to account for repeated measures. Additionally, the ‘circacompare’ package was used to investigate and compare the rhythms of infected and uninfected individuals in 12:12 h light: dark regime over a 48 h period. For experiment 2, 1 GLMM, fitted with ‘binomial error’ family and ‘logit’ link function, was used to assess diel activity patterns of infected and uninfected guppies. In the GLMM, the proportion of time fish remained actively swimming during a 5-min period was the dependent term in the model, and fixed effects included infection status (infected or uninfected) and ZT as well as an interaction term between infection status and ZT, and the shoal number was included as a random term to account for repeated measures. For experiment 3, a Generalized Linear Model (GLM) was used to compare parasite activity (number of probes) with respect to different ZT and light conditions. An interaction term between ZT and light conditions was incorporated into the model. Moreover, the ‘circacompare’ package was used to investigate and compare rhythms of parasite activity in different light conditions. For experiment 4, 2 GLMs, fitted with ‘binomial error’ family and ‘logit’ link function assessed the proportion of parasites transmitting from an infected host to its uninfected conspecific and proportion of parasites that had been dislodged with respect to ZT, light conditions and parasite density on the host. In all tests, the level of significance was taken as *P* < 0.05. GLMM models were refined through stepwise deletion of non-significant terms and AIC comparisons and their robustness was assessed using residual plots, indicating that assumptions of models were met (Pinheiro and Bates, 2000). Mean standard length was not included within models, as fish were size-matched at the start of each experiment to eliminate size variability.

## Results

Overall, the circadian rhythm detected in guppy activity was disturbed by infection, resulting in increased activity at night, thus nocturnal restlessness both in isolated guppies and in shoals. Even though gyrodactylid behaviour and activity did not exhibit diurnal variance, parasite activity peaked at night, coinciding with the increase in host activity.

### Experiment 1: impact of infection on daily activity of isolated guppies

**Figure 3.**
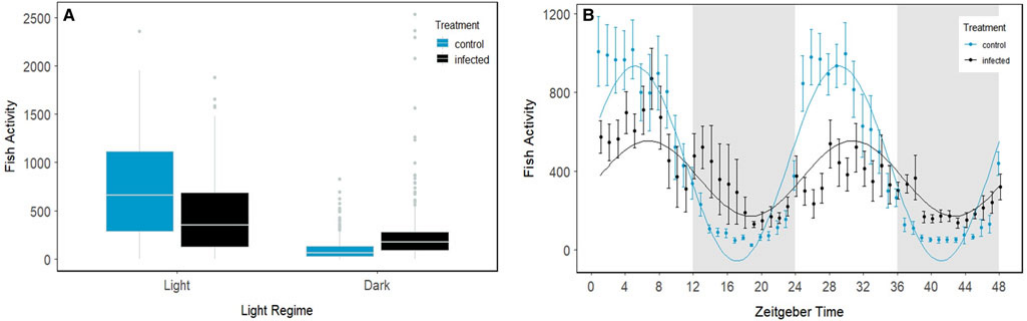
(a). Activity of isolated guppies from uninfected and *Gyrodactylus turnbulli* infected fish in light and dark conditions. In the light, the uninfected guppies were significantly more active than the infected fish and in the dark uninfected guppies were significantly less active than their infected conspecifics. Fish activity is defined as the number of times fish went through the infrared beams per tank, as retrieved from the TriKinetics software. Dots represent outliers; the box the first and third quartile with median and the line 50% of fish activity. (b). The activity of uninfected and infected guppies monitored hourly for a 47 h period. Grey areas indicate dark periods. Error bars represent standard error.

For both uninfected (control) and infected guppies there was a significant difference in activity between light and dark conditions (emmeans; df = 1; *P* < 0.0001 and *P* < 0.0001 respectively; Fig. 3a), with both uninfected and infected fish having significantly higher activity in the light comparing to the dark conditions. In light conditions, uninfected guppies were significantly more active than infected guppies (emmeans; df = 1; *P* = 0.0005), whilst the opposite was observed in dark conditions, whereby uninfected guppies were less active than their infected conspecifics (emmeans; df = 1; *P* = 0.036; Fig. 3a). The ‘circacompare’ package confirmed the presence of circadian rhythmicity in activity of both the uninfected (*P* = 0.006) and infected group (*P* = 0.0008; Fig. 3b). The 2 rhythms had a significant difference in MESOR (*P* = 0.0008), with the uninfected group having a greater rhythm-adjusted mean than infected group, in acrophase (*P* = 0.0004) with the uninfected group having an earlier peak and a significantly higher amplitude, which is the half of the predictable variation in activity throughout the 48 h period (*P*���= 0.0002; Fig. 3b).

### Experiment 2: impact of infection on daily activity in guppy shoals

**Figure 4.**
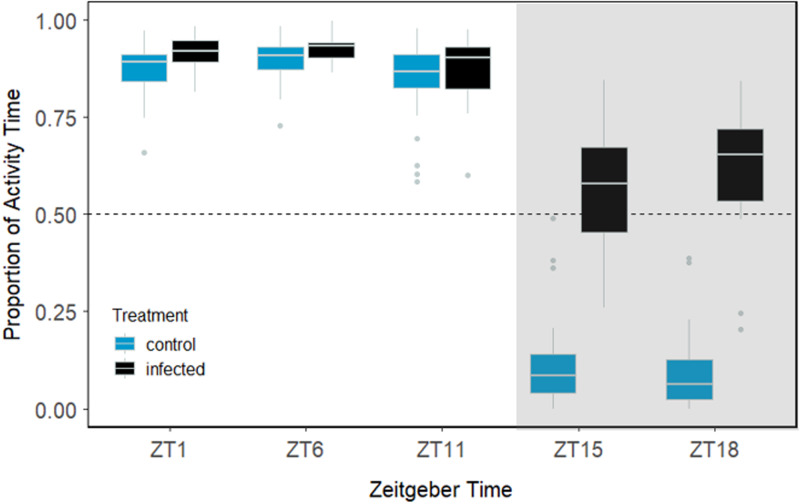
The proportion of time *Gyrodactylus turnbulli* infected and uninfected guppies remained active during 5-min focal follows at 5 ZT timepoints. Grey areas indicate dark periods. Black dots represent outliers; bars the upper and lower limits; the box the first and third quartile with median, and the dashed line 50% of the time in which guppies remained active during a focal follow.

Swimming activity of uninfected guppies was elevated during the day and dropped drastically at night. When guppies were infected, however, they exhibited nocturnal restlessness with increased swimming activity, indicating that infection status had a significant effect on swimming activity of guppies when in shoals, which also depended on ZT (ZT × Infection status interaction; GLMM; *P* < 0.001). When studying shoal swimming activity at specific ZT timepoints, uninfected guppies were significantly less active than infected conspecifics at each timepoint (GLMM; df = 4; *P* < 0.001), evidently more so during nocturnal hours where there is a great difference in activity of uninfected and infected shoaling guppies (ZT15, ZT18; Fig. 4).

### Experiment 3: impact of photoperiod on parasite daily activity

**Figure 5.**
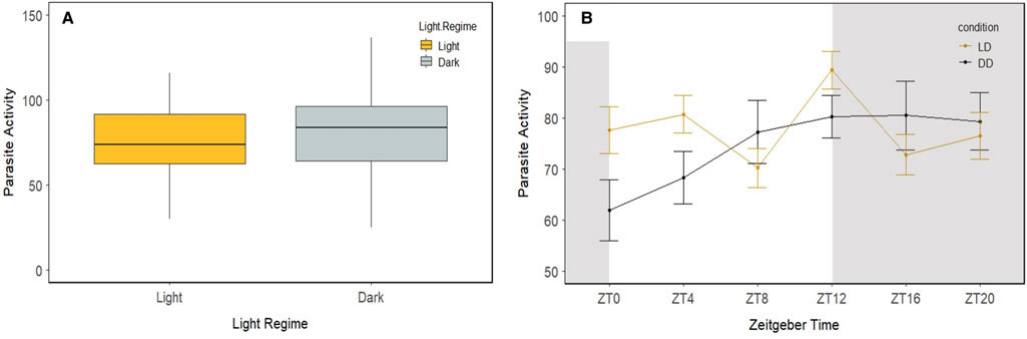
(a). Activity (number of probes) of *Gyrodactylus turnbulli* parasites present on their guppy host in light and dark conditions. The box represents the first and third quartile with median. (b). Parasite activity recorded both in 12:12 h light: dark regime (LD) and 48 h constant darkness (DD). There was significant difference in activity at ZT0, ZT4, ZT8, ZT12 and ZT16. However, there was no rhythmicity detected in either case. Grey areas indicate dark periods. Error bars represent standard error.

Light conditions and ZT timepoint both had a significant effect on parasite activity (GLM; *P* = 0.007 and *P* < 0.001 respectively) as well as their interaction (Light conditions × ZT timepoints; GLM; *P* < 0.001). Overall, parasites were more active in the dark compared to light conditions under the 12:12 h light: dark regime (GLM; df = 1; *P* = 0.0004; Fig. 5a). When comparing parasite activity between the 12:12 h light: dark regime and constant darkness (Fig. 5b), there was a significant difference in ZT0 (emmeans; df = 1; *P* < 0.0001), ZT4 (emmeans; df = 1; *P* < 0.0001), ZT8 (emmeans; df = 1; *P* = 0.015), ZT12 (emmeans; df = 1; *P* = 0.004), ZT16 (emmeans; df = 1; *P* = 0.009) but not ZT20 (emmeans; df = 1; *P* = 0.342). The ‘circacompare’ package, however, did not detect a circadian rhythm in parasite activity either in 12:12 h light: dark or 48 h of darkness regime, suggesting that it is not endogenously driven, but affected by other cues (Fig. 5b).

**Figure 6.**
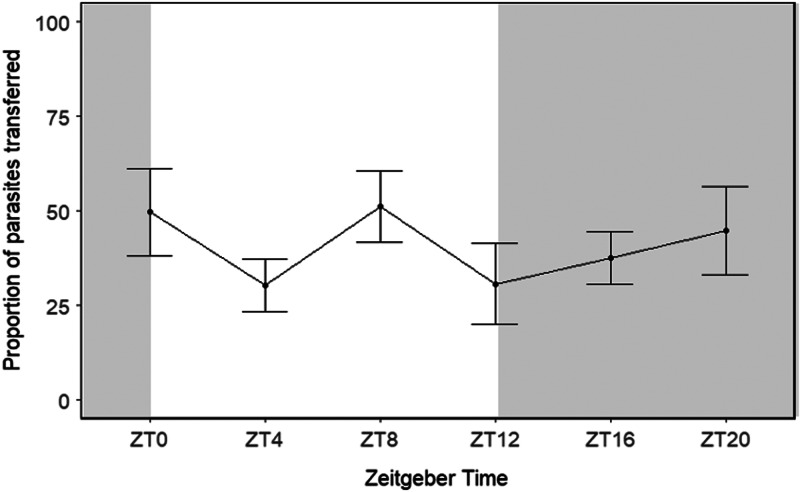
Proportion of parasites that transferred from the host to the recipient conspecific at different ZT points in a 12:12 h light: dark regime with no significant difference recorded. Grey areas indicate dark periods. Error bars represent standard error.

### Experiment 4: impact of photoperiod on parasite daily transmissibility

The proportion of parasites that transferred from an infected host to an uninfected conspecific (GLMM; df = 5; *P* > 0.05) or the proportion of parasites that dislodged from their host (GLMM; df = 5; *P* > 0.05) were not significantly different between ZT timepoints, in light *vs* dark conditions or dependent on parasite density of the host. Also, the ‘circacompare’ package did not detect a rhythm in parasite transmissibility in the 12:12 h light: dark regime (*P* > 0.05) with no significant difference detected in MESOR, amplitude and phase.

## Discussion

Here, we provide the first empirical evidence of aquatic ectoparasites directly altering ‘rest–activity’ cycles of diurnal fish hosts. Using the guppy-gyrodactylid system, we showed infection changes the daily rhythms of guppy activity; infected individuals were more active at night than their uninfected conspecifics, with nocturnal restlessness exhibited both in isolated and guppy shoals. Although gyrodactylid behaviour (host-searching activity and transmissibility) did not exhibit diurnal cycles, parasites did display elevated activity at night (supporting Brooker *et al*., 2011). Our results are important because regulated rest–activity cycles are essential for physical and mental wellbeing (Besedovsky *et al*., 2012) and most notably optimizing efficient immune functioning (Imeri and Opp, 2009). Sleep deprivation can result in cognitive impairment (Alhola and Polo-Kantola, 2007) and increased disease susceptibility (Cohen *et al*., 2009). Moreover, disease itself induces dramatic sleep alterations, although previously only reported for endoparasite infections (Norman *et al*., 1990; Buguet *et al*., 1993; Toth, 1995).

Ectoparasites likely inflict some degree of physical discomfort to their hosts during establishment on the host skin and throughout infection. In the case of gyrodactylids, they attach to their host primarily using hooks, and following establishment extrude digestive enzymes onto the hosts' skin from which host epidermal cells and mucus are subsequently ingested (Bakke *et al*., 2007). The frequent movement of gyrodactylids across the host's skin, potentially associated with their avoidance of localized host immune responses (Richards and Chubb, 1996), may irritate the guppy hosts and result in increased host nocturnal activity. In the case of host activity experiments, both when isolated and in shoals, observations started within 24 h of parasite infection, so shortly after host infection. As also shown in other parasite species, the brain-infecting *Euhaplorchis californiensis* cercariae had an impact on their killifish host (*Fundulus parvipinnis*) during parasite exposure. Host activity and metabolic rate increased, with metabolic rate remaining elevated while activity returned to normal, suggesting ongoing physiological changes are separate from behavioural effects (Nadler *et al*., 2021). So, migration of gyrodactylids across the host's skin and subsequent irritation may have been a driver of host nocturnal restlessness.

Whilst not measured here, complex interactions between immune and hormonal modulation activated by infection may have also contributed to nocturnal restlessness. Inflammatory responses to infection, for example, significantly contribute to sleep disturbances (Ali *et al*., 2013). In fish, a typical response to ectoparasite infection is epidermal thickening (Esteban *et al*., 2012; Smallbone *et al*., 2016), whereby inflammation at the site of parasite establishment occurs after tegument damage (Lindenstrøm *et al*., 2004). Inflammatory responses are regulated by pro- and anti-inflammatory cytokines, which promote and inhibit rest, respectively. The production and release of pro-inflammatory cytokines has been associated with the secretion of melatonin (Srinivasan *et al*., 2005): a regulatory hormone essential for enhancing propensity to sleep (Zhdanova *et al*., 2001; Cajochen *et al*., 2003). Perturbances in natural oscillations of a protein like melatonin can promote restlessness (Budh *et al*., 2005). So, disease can induce dramatic rest alterations, so far only emphasized in endoparasite infections (Norman *et al*., 1990; Buguet *et al*., 1993; Toth, 1995), leading to a constant state of restlessness. However, previous studies have shown that shortly after infection, effective localized immune responses towards gyrodactylids and infection-related changes in gene expression are exhibited (Lindenstrøm *et al*., 2003; Bakke *et al*., 2007; Konczal *et al*., 2020). Therefore, these disruptions in immune responses could further promote host restlessness. Overall, there is increasing evidence of complex interactions between molecular clocks and immunity (Ellison *et al*., 2021), as dysregulation of certain host clock proteins linked with cell function, defence and inflammation may lead, among others, to inflammatory diseases and immunodeficiency (Curtis *et al*., 2014). While clock gene expression drives daily cycles in immunity, immune activation caused by infection can itself alter clock gene expression. Therefore, we suggest the observed changes in daily behaviour patterns could a result of altered clocks.

Regarding parasite activity, even though no ‘true’ circadian rhythmicity in phenotypes was detected, host seeking behaviour and activity were greater in the dark and more specifically at the end of the light period (ZT12), where there was a peak in parasite activity. Interestingly, this elevated host seeking behaviour coincides with natural guppy shoaling behaviour peaking around dusk (Croft *et al*., 2003; O'Connor and Krause, 2003). Thus, an increase in parasite activity could potentially facilitate transmission between hosts, as fish are closely aggregated during shoaling (Pitcher, 1983). Elevated host activity may be beneficial to individuals as infected fish move between and directly contact resting conspecifics (Reynolds *et al*., 2019), potentially diluting their parasite burdens (Mooring and Hart, 1992). Previous studies demonstrate the benefits of successful parasite transmission in terms of ‘vaccinating’ hosts against subsequent infections (Faria *et al*., 2010), but also reducing resource competition between parasites and allowing short-term evasion of a hosts' immune response (Richards and Chubb, 1996), concluding that a driver of parasite activity could be host behaviour.

Better understanding of both guppy and gyrodactylid behavioural and activity patterns, provides a greater insight in host–parasite dynamics. Using this knowledge of circadian rhythms may be helpful in tackling infectious diseases, as chronotherapeutic approaches could be used to yield maximum therapeutant efficiency based on host metabolism, when treating for parasites. In aquaculture, parasite infections do not always lead to fish mortality, yet still negatively impact productivity, health and welfare of fish (Shinn *et al*., 2015), thus extensive use of therapeutics is used to maximize efficiency (Burka *et al*., 1997; Grant, 2002). However, drug efficacy and toxicity vary with time of day (Bruguerolle, 1998), as daily rhythms in drug absorption, metabolism, detoxification and excretion have been reported in mammalian species (Smolensky and Peppas, 2007). As shown by Vera and Migaud (2016).

Atlantic salmon (*S. salar*) treated with hydrogen peroxide (H_2_O_2_) revealed increased sublethal toxic effect during the first half of the day, providing the first evidence of chronotoxicity in Atlantic salmon. Moreover, the impact of photoperiod and infection status on immune gene activation as well as immune expression and rhythmicity was investigated by Ellison *et al*. (2021), where it was shown that circadian perturbation, that shifts the magnitude and timing of immune activity, is detrimental to fish health. These studies provide evidence for potential optimization of treatment timing in aquaculture, opening the door to treating fish diseases chronotherapeutically. In addition, non-detected infections, which alter fish behaviour such as increased restlessness, could be used as a diagnostic tool for emerging infectious diseases.

In conclusion, we demonstrate that ectoparasites alter daily rhythmic activity of their hosts, resulting in greater nocturnal restlessness both individually and in shoals. Circadian rhythmicity in activity was present and distinctly different between uninfected and infected fish. Peaks in activity may be driven parasite skin irritation as well as immune responses to infection, such as infection resolution and repair, which are elevated at night (Ellison *et al*., 2021) and may have direct implications for other animal behaviour studies that overlook nocturnal activity of diurnal species. We also provide a better understanding of parasite behaviour, that does not exhibit a daily rhythmical variance, but peaks in the dark, coinciding with infected fish behaviour. As gyrodactylids pose a significant threat to many economically important fish in aquaculture (Lafferty *et al*., 2015; Shinn *et al*., 2015), the use and application of chronotherapy to maximize treatment efficacy could be a potential solution to this problem.

## Data Availability

The data that support the findings of this study are openly available in Mendeley at http://doi.org/10.17632/p23ghwjhxj.1 Arapi, Elissavet (2024), “Restless nights when sick: ectoparasite infections alter rest-activity cycles of diurnal fish hosts”, Mendeley Data, V2, doi: 10.17632/p23ghwjhxj.1

## References

[ref1] Adam K (1980) Sleep as a restorative process and a theory to explain why. Progress in Brain Research 53, 289–305. 7005947 10.1016/S0079-6123(08)60070-9

[ref2] Adams KL, Castanon-Cervantes O, Evans JA and Davidson AJ (2013) Environmental circadian disruption elevates the IL-6 response to lipopolysaccharide in blood. Journal of Biological Rhythms 28, 272–277. 23929554 10.1177/0748730413494561 PMC4097096

[ref3] Alhola P and Polo-Kantola P (2007) Sleep deprivation: impact on cognitive performance. Neuropsychiatric Disease and Treatment 3, 553–567. 19300585 PMC2656292

[ref4] Ali T, Choe J, Awab A, Wagener TL and Orr WC (2013) Sleep, immunity and inflammation in gastrointestinal disorders. World Journal of Gastroenterology: WJG 19, 9231. 24409051 10.3748/wjg.v19.i48.9231 PMC3882397

[ref5] Bakke TA, Cable J and Harris PD (2007) The biology of gyrodactylid monogeneans: the ‘Russian-doll killers’. Advances in Parasitology 64, 161–460. 17499102 10.1016/S0065-308X(06)64003-7

[ref6] Bass J and Lazar MA (2016) Circadian time signatures of fitness and disease. Science 354, 994–999. 27885004 10.1126/science.aah4965

[ref7] Bates D, Mächler M, Bolker B and Walker S (2015) Fitting linear mixed-effects models using lme4. Journal of Statistical Software 67, 1–48.

[ref8] Benington JH and Craig Heller H (1995) Restoration of brain energy metabolism as the function of sleep. Progress in Neurobiology 45, 347–360. 7624482 10.1016/0301-0082(94)00057-o

[ref9] Besedovsky L, Lange T and Born J (2012) Sleep and immune function. Pflügers Archiv-European Journal of Physiology 463, 121–137. 22071480 10.1007/s00424-011-1044-0 PMC3256323

[ref10] Boeuf G and Le Bail PY (1999) Does light have an influence on fish growth? Aquaculture 177, 129–152.

[ref11] Brooker AJ, Grano Maldonado MI, Irving S, Bron JE, Longshaw M and Shinn AP (2011) The effect of octopaminergic compounds on the behaviour and transmission of *Gyrodactylus*. Parasites & Vectors 4, 207. 22032413 10.1186/1756-3305-4-207 PMC3212917

[ref12] Brown MA, Quan SF and Eichling PS (2011) Circadian rhythm sleep disorder, free-running type in a sighted male with severe depression, anxiety, and agoraphobia. Journal of Clinical Sleep Medicine: JCSM: Official Publication of the American Academy of Sleep Medicine 7, 93–94. 21344043 PMC3041617

[ref13] Bruguerolle B (1998) Chronopharmacokinetics: current Status. Clinical Pharmacokinetics 35, 83–94. 9739478 10.2165/00003088-199835020-00001

[ref14] Bryant PA, Trinder J and Curtis N (2004) Sick and tired: does sleep have a vital role in the immune system? Nature Reviews Immunology 4, 457–467. 10.1038/nri1369 15173834

[ref15] Budh CN, Hultling C and Lundeberg T (2005) Quality of sleep in individuals with spinal cord injury: a comparison between patients with and without pain. Spinal Cord 43, 85–95. 15570322 10.1038/sj.sc.3101680

[ref16] Buguet A, Bert J, Tapie P, Tabaraud F, Doua F, Lonsdorfer J, Bogui P and Dumas M (1993) Sleep-wake cycle in human African trypanosomiasis. Journal of Clinical Neurophysiology: Official Publication of the American Electroencephalographic Society 10, 190–196. 8389383 10.1097/00004691-199304000-00006

[ref17] Burka JF, Hammell KL, Horsberg TE, Johnson GR, Rainnie DJ and Speare DJ (1997) Drugs in salmonid aquaculture – a review. Journal of Veterinary Pharmacology and Therapeutics 20, 333–349. 9350253 10.1046/j.1365-2885.1997.00094.x

[ref18] Cable J and van Oosterhout C (2007) The impact of parasites on the life history evolution of guppies (*Poecilia reticulata*): the effects of host size on parasite virulence. International Journal for Parasitology 37, 1449–1458. 17561023 10.1016/j.ijpara.2007.04.013

[ref19] Cable J, Scott ECG, Tinsley RC and Harris PD (2002) Behavior favoring transmission in the viviparous monogenean *Gyrodactylus turnbulli*. Journal of Parasitology 88, 183–184. 12053961 10.1645/0022-3395(2002)088[0183:BFTITV]2.0.CO;2

[ref20] Cajochen C, Kräuchi K and Wirz-Justice A (2003) Role of melatonin in the regulation of human circadian rhythms and sleep: melatonin, sleep and circadian rhythms. Journal of Neuroendocrinology 15, 432–437. 12622846 10.1046/j.1365-2826.2003.00989.x

[ref21] Castanon-Cervantes O, Wu M, Ehlen JC, Paul K, Gamble KL, Johnson RL, Besing RC, Menaker M, Gewirtz AT and Davidson AJ (2010) Dysregulation of inflammatory responses by chronic circadian disruption. The Journal of Immunology 185, 5796–5805. 20944004 10.4049/jimmunol.1001026 PMC2974025

[ref22] Chambel J, Pinho R, Sousa R, Ferreira T, Baptista T, Severiano V, Mendes S and Pedrosa R (2015) The efficacy of MS-222 as anaesthetic agent in four freshwater aquarium fish species. Aquaculture Research 46, 1582–1589.

[ref23] Cirelli C and Tononi G (2008) Is sleep essential? PLoS Biology 6, e216. 18752355 10.1371/journal.pbio.0060216 PMC2525690

[ref24] Cohen S, Doyle WJ, Alper CM, Janicki-Deverts D and Turner RB (2009) Sleep habits and susceptibility to the common cold. Archives of Internal Medicine 169, 62. 19139325 10.1001/archinternmed.2008.505 PMC2629403

[ref25] Croft DP, Arrowsmith BJ, Bielby J, Skinner K, White E, Couzin ID, Magurran AE, Ramnarine I and Krause J (2003) Mechanisms underlying shoal composition in the Trinidadian guppy, *Poecilia reticulata*. Oikos 100, 429–438.

[ref26] Curtis AM, Bellet MM, Sassone-Corsi P and O'Neill LAJ (2014) Circadian clock proteins and immunity. Immunity 40, 178–186. 24560196 10.1016/j.immuni.2014.02.002

[ref27] Davidson AJ (2006) Search for the feeding-entrainable circadian oscillator: a complex proposition. American Journal of Physiology-Regulatory, Integrative and Comparative Physiology 290, R1524–R1526. 16455773 10.1152/ajpregu.00073.2006

[ref28] Doube BM (1975) Cattle and the paralysis tick *Ixodes holocyclus*. Australian Veterinary Journal 51, 511–515. 1220655 10.1111/j.1751-0813.1975.tb06901.x

[ref29] DuRant SE, Hopkins WA, Davis AK and Romero LM (2015) Evidence of ectoparasite-induced endocrine disruption in an imperilled giant salamander, the eastern hellbender (*Cryptobranchus alleganiensis*). Journal of Experimental Biology 218, 2297–2304. 26034123 10.1242/jeb.118703

[ref30] Ellison AR, Wilcockson D and Cable J (2021) Circadian dynamics of the teleost skin immune-microbiome interface. Microbiology 9, 1–18. 10.1186/s40168-021-01160-4 PMC8594171 34782020

[ref31] Ernst I and Whittington ID (1996) Hatching rhythms in the capsalid monogeneans *Benedenia lutjani* from the skin and *B. rohdei* from the gills of *Lutjanus carponotatus* at Heron Island, Queensland, Australia. International Journal for Parasitology 26, 1191–1204. 9024862 10.1016/s0020-7519(96)00118-x

[ref32] Esteban M, Castaño A, Schindler B, Koch H, Angerer J, Casteleyn L, Joas R, Joas A, Biot P, Aerts D and Kolossa-Gehring M (2012) S-150: strategies and tools to obtain comparable biomarker results in the Pan-European COPHES human biomonitoring project. Epidemiology 23.

[ref33] Faria PJ, van Oosterhout C and Cable J (2010) Optimal release strategies for captive-bred animals in reintroduction programs: experimental infections using the guppy as a model organism. Biological Conservation 143, 35–41.

[ref34] Gannicott AM and Tinsley RC (1997) Egg hatching in the monogenean gill parasite *Discocotyle sagittata* from the rainbow trout (*Oncorhynchus mykiss*). Cambridge University Press 114, 569–579.

[ref35] Grant AN (2002) Medicines for sea lice. Pest Management Science 58, 521–527. 12138618 10.1002/ps.481

[ref36] Griffiths S W and Magurran A E (1997) Familiarity in schooling fish: how long does it take to acquire? Animal Behaviour 53(5), 945–949.

[ref37] Harris PD, Shinn AP, Cable J and Bakke TA (2004) Nominal species of the genus *Gyrodactylus* von Nordmann 1832 (Monogenea: Gyrodactylidae), with a list of principal host species. Systematic Parasitology 59, 1–27. 15318017 10.1023/B:SYPA.0000038447.52015.e4

[ref38] Helvig A, Wade S and Hunter-Eades L (2016) Rest and the associated benefits in restorative sleep: a concept analysis. Journal of Advanced Nursing 72, 62–72. 26370516 10.1111/jan.12807

[ref39] Ibarra-Coronado EG, Pantaleón-Martínez AM, Velazquéz-Moctezuma J, Prospéro-García O, Méndez-Díaz M, Pérez-Tapia M, Pavón L and Morales-Montor J (2015) The bidirectional relationship between sleep and immunity against infections. Journal of Immunology Research 2015, 1–14. 10.1155/2015/678164 PMC4568388 26417606

[ref40] Imeri L and Opp MR (2009) How (and why) the immune system makes us sleep. Nature Reviews Neuroscience 10, 199–210. 19209176 10.1038/nrn2576 PMC2839418

[ref41] Karatsoreos I N and Silver R (2017) Body clocks in health and disease. In Conn's Translational Neuroscience. Cambridge, Massachusetts: Academic Press, pp. 599–615.

[ref42] Kearn GC (1967) Experiments on host-finding and host-specificity in the monogenean skin parasite *Entobdella soleae*. Parasitology 57, 585–605. 6069119 10.1017/s0031182000072450

[ref43] Kilkenny C, Browne W, Cuthill I, Emerson M and Altman D (2014) Improving bioscience research reporting: the ARRIVE guidelines for reporting animal research. Animals 4, 35–44. 10.1016/j.joca.2012.02.010 22424462

[ref44] Kolluru GR, Grether GF, Dunlop E and South SH (2009) Food availability and parasite infection influence mating tactics in guppies (*Poecilia reticulata*). Behavioural Ecology 20, 131–137.

[ref45] Konczal M, Ellison AR, Phillips KP, Radwan J, Mohammed RS, Cable J and Chadzinska M (2020) RNA-Seq analysis of the guppy immune response against *Gyrodactylus bullatarudis* infection. Parasite Immunology 42. 10.1111/pim.12782 32738163

[ref46] Kripke DF, Garfinkel L, Wingard DL, Klauber MR and Marler MR (2002) Mortality associated with sleep duration and insomnia. Archives of General Psychiatry 59, 131. 11825133 10.1001/archpsyc.59.2.131

[ref47] Krueger JM, Frank MG, Wisor JP and Roy S (2016) Sleep function: toward elucidating an enigma. Sleep Medicine Reviews 28, 46–54. 26447948 10.1016/j.smrv.2015.08.005 PMC4769986

[ref48] Lafferty KD, Harvell CD, Conrad JM, Friedman CS, Kent ML, Kuris AM, Powell EN, Rondeau D and Saksida SM (2015) Infectious diseases affect marine fisheries and aquaculture economics. Annual Review of Marine Science 7, 471–496. 10.1146/annurev-marine-010814-015646 25251276

[ref49] Leung LC, Wang GX, Madelaine R, Skariah G, Kawakami K, Deisseroth K, Urban AE and Mourrain P (2019) Neural signatures of sleep in zebrafish. Nature 571, 198–204. 31292557 10.1038/s41586-019-1336-7 PMC7081717

[ref50] Liang X, Bushman FD and FitzGerald GA (2015) Rhythmicity of the intestinal microbiota is regulated by gender and the host circadian clock. Proceedings of the National Academy of Sciences 112, 10479–10484. 10.1073/pnas.1501305112 PMC4547234 26240359

[ref51] Lindenstrøm T, Buchmann K and Secombes CJ (2003) *Gyrodactylus derjavini* infection elicits IL-1*β* expression in rainbow trout skin. Fish & Shellfish Immunology 15, 107–115. 12834615 10.1016/s1050-4648(02)00142-0

[ref52] Lindenstrøm T, Secombes CJ and Buchmann K (2004) Expression of immune response genes in rainbow trout skin induced by *Gyrodactylus derjavini* infections. Veterinary Immunology and Immunopathology 97, 137–148. 14741133 10.1016/j.vetimm.2003.08.016

[ref53] Majde J and Krueger J (2005) Links between the innate immune system and sleep. Journal of Allergy and Clinical Immunology 116, 1188–1198. 16337444 10.1016/j.jaci.2005.08.005

[ref54] Mideo N, Reece SE, Smith AL and Metcalf CJE (2013) The Cinderella syndrome: why do malaria-infected cells burst at midnight? Trends in Parasitology 29, 10–16. 23253515 10.1016/j.pt.2012.10.006 PMC3925801

[ref55] Midttun HL, Vindas MA, Whatmore PJ, Øverli Ø and Johansen IB (2020) Effects of *Pseudoloma neurophilia* infection on the brain transcriptome in zebrafish (*Danio rerio*). Journal of Fish Diseases 43, 863–875. 32542843 10.1111/jfd.13198

[ref56] Mooring MS and Hart BL (1992) Animal grouping for protection from parasites: selfish herd and encounter-dilution effects. Behaviour 123, 173–193.

[ref57] Nadler LE, Bengston E, Eliason EJ, Hassibi C, Helland-Riise SH, Johansen IB, Kwan GT, Tresguerres M, Turner AV, Weinersmith KL and Øverli Ø (2021) A brain-infecting parasite impacts host metabolism both during exposure and after infection is established. Functional Ecology 35, 105–116.

[ref58] Norman SE, Chediak AD, Kiel M and Cohn MA (1990) Sleep disturbances in HIV-infected homosexual men. AIDS 4, 775–782. 2261133 10.1097/00002030-199008000-00009

[ref59] O'Connor E and Krause J (2003) Effect of light intensity on the shoaling behaviour of the guppy (*Poecilia reticulata*). Journal of Fish Biology 63, 254–254.

[ref60] O'Donnell AJ, Schneider P, McWatters HG and Reece SE (2011) Fitness costs of disrupting circadian rhythms in malaria parasites. Proceedings of the Royal Society B: Biological Sciences 278, 2429–2436. 10.1098/rspb.2010.2457 PMC3125626 21208950

[ref61] Opp MR (2009) Sleep and psychoneuroimmunology. Immunology and Allergy Clinics of North America 29, 295–307. 19389583 10.1016/j.iac.2009.02.009

[ref62] Otsuka K, Cornelissen G, Furukawa S, Kubo Y, Hayashi M, Shibata K, Mizuno K, Aiba T, Ohshima H and Mukai C (2016) Long-term exposure to space's microgravity alters the time structure of heart rate variability of astronauts. Heliyon 2, e00211. 28050606 10.1016/j.heliyon.2016.e00211 PMC5192238

[ref63] Parsons R, Parsons R, Garner N, Oster H and Rawashdeh O (2020) CircaCompare: a method to estimate and statistically support differences in mesor, amplitude and phase, between circadian rhythms. Valencia, A. ed. Bioinformatics 36, 1208–1212. 31588519 10.1093/bioinformatics/btz730

[ref64] Penev PD, Kolker DE, Zee PC and Turek FW (1998) Chronic circadian desynchronization decreases the survival of animals with cardiomyopathic heart disease. American Journal of Physiology-Heart and Circulatory Physiology 275, H2334–H2337. 10.1152/ajpheart.1998.275.6.H2334 9843836

[ref65] Piggins HD (2002) Human clock genes. Annals of Medicine 34, 394–400. 12452483 10.1080/078538902320772142

[ref66] Pinheiro JC and Bates DM (2000) Extending the basic linear mixed-effects model. In Mixed-Effects Models in S and S-PLUS. Statistics and Computing. New York: Springer-Verlag, pp. 201–270.

[ref67] Pitcher TJ (1983) Heuristic definitions of shoaling behaviour. Animal Behaviour 31, 611–163.

[ref68] Preston BT, Capellini I, McNamara P, Barton RA and Nunn CL (2009) Parasite resistance and the adaptive significance of sleep. BMC Evolutionary Biology 9, 7. 19134175 10.1186/1471-2148-9-7 PMC2631508

[ref69] R Core Team (2019) R: A Language and Environment for Statistical Computing. Vienna, Austria: R Foundation for Statistical Computing.

[ref70] Reebs SG (2002) Plasticity of diel and circadian activity rhythms in fishes. Reviews in Fish Biology and Fisheries 12, 349–371.

[ref71] Reynolds M, Hockley FA, Wilson CAME and Cable J (2019) Assessing the effects of water flow rate on parasite transmission amongst a social host. Hydrobiologia 830, 201–212.

[ref72] Richards GR and Chubb JC (1996) Host response to initial and challenge infections, following treatment, of *Gyrodactylus bullatarudis* and *G. turnbulli* (Monogenea) on the guppy (*Poecilia reticulata*). Parasitology Research 82, 242–247. 8801557 10.1007/s004360050103

[ref73] Rijo-Ferreira F, Pinto-Neves D, Barbosa-Morais NL, Takahashi JS and Figueiredo LM (2017) *Trypanosoma brucei* metabolism is under circadian control. Nature Microbiology 2, 17032. 10.1038/nmicrobiol.2017.32 PMC5398093 28288095

[ref74] Rinaldi L, Russo T, Schioppi M, Pennacchio S and Cringoli G (2007) *Passalurus ambiguus*: new insights into copromicroscopic diagnosis and circadian rhythm of egg excretion. Parasitology Research 101, 557–561. 17372763 10.1007/s00436-007-0513-z

[ref75] Schelkle B, Shinn A, Peeler E and Cable J (2009) Treatment of gyrodactylid infections in fish. Diseases of Aquatic Organisms 86, 65–75. 19899351 10.3354/dao02087

[ref76] Searle SR, Speed FM and Milliken GA (1980) Population marginal means in the linear model: an alternative to least squares means. The American Statistician 34, 216–221.

[ref77] Shinn AP, Pratoomyot J, Bron J, Paladini G, Brooker EE and Brooker AJ (2015) Economic impacts of aquatic parasites on global finfish production. Global Aquaculture Advocate 2015, 58–61.

[ref78] Siegel JM (2008) Do all animals sleep? Trends in Neurosciences 31, 208–213. 18328577 10.1016/j.tins.2008.02.001 PMC8765194

[ref79] Smallbone W, van Oosterhout C and Cable J (2016) The effects of inbreeding on disease susceptibility: *Gyrodactylus turnbulli* infection of guppies, *Poecilia reticulata*. Experimental Parasitology 167, 32–37. 27130704 10.1016/j.exppara.2016.04.018

[ref80] Smolensky MH and Peppas NA (2007) Chronobiology, drug delivery, and chronotherapeutics. Advanced Drug Delivery Reviews 59, 828–851. 17884237 10.1016/j.addr.2007.07.001

[ref81] Sollars PJ and Pickard GE (2015) The neurobiology of circadian rhythms. Psychiatric Clinics of North America 38, 645–665. 26600101 10.1016/j.psc.2015.07.003 PMC4660252

[ref82] Srinivasan V, Maestroni GJ, Cardinali DP, Esquifino AI, Perumal SR and Miller SC (2005) Melatonin, immune function and aging. Immunity & Ageing 2, 1–10. 16316470 10.1186/1742-4933-2-17 PMC1325257

[ref83] Stewart A, Jackson J, Barber I, Eizaguirre C, Paterson R, van West P, Williams C and Cable J (2017) Hook, line and infection: a guide to culturing parasites, establishing infections and assessing immune responses in the three-spined stickleback. Advances in Parasitology 98, 39–109. 28942772 10.1016/bs.apar.2017.07.001

[ref84] Takahashi K, Lin JS and Sakai K (2008) Neuronal activity of orexin and non-orexin waking-active neurons during wake–sleep states in the mouse. Neuroscience 153, 860–870. 18424001 10.1016/j.neuroscience.2008.02.058

[ref85] Taranger GL, Carrillo M, Schulz RW, Fontaine P, Zanuy S, Felip A, Weltzien FA, Dufour S, Karlsen Ø, Norberg B and Andersson E (2010) Control of puberty in farmed fish. General and Comparative Endocrinology 165, 483–515. 19442666 10.1016/j.ygcen.2009.05.004

[ref86] Theron A and Combes C (1995) Asynchrony of infection timing, habitat preference, and sympatric speciation of schistosome parasites. Evolution 49, 372. 28565017 10.1111/j.1558-5646.1995.tb02249.x

[ref87] Toth LA (1995) Sleep, sleep deprivation and infectious disease: studies in animals. Advances in Neuroimmunology 5, 79–92. 7795895 10.1016/0960-5428(94)00045-p

[ref88] Van Oosterhout C, Harris PD and Cable J (2003) Marked variation in parasite resistance between two wild populations of the Trinidadian guppy, *Poecilia reticulata* (Pisces: Poeciliidae): parasite resistance in the Trinidadian guppy. Biological Journal of the Linnean Society 79, 645–651.

[ref89] Vera LM and Migaud H (2016) Hydrogen peroxide treatment in Atlantic salmon induces stress and detoxification response in a daily manner. Chronobiology International 33, 530–542. 27058450 10.3109/07420528.2015.1131164

[ref90] Whiting JR, Mahmud MA, Bradley JE and MacColl ADC (2020) Prior exposure to long-day photoperiods alters immune responses and increases susceptibility to parasitic infection in stickleback. Proceedings of the Royal Society B: Biological Sciences 287, 20201017. 10.1098/rspb.2020.1017 PMC7423467 32605431

[ref91] Wickham H (2009) Getting started with qplot. In ggplot2. Use R. New York, NY: Springer, pp. 9–26.

[ref92] Widder EA, Robison BH, Reisenbichler KR and Haddock SHD (2005) Using red light for *in situ* observations of deep-sea fishes. Deep Sea Research Part I: Oceanographic Research Papers 52, 2077–2085.

[ref93] Zhdanova IV, Wang SY, Leclair OU and Danilova NP (2001) Melatonin promotes sleep-like state in zebrafish. Brain Research 903, 263–268. 11382414 10.1016/s0006-8993(01)02444-1

[ref94] Zietara MS and Lumme J (2002) Speciation by host switch and adaptive radiation in a fish parasite genus *Gyrodactylus* (monogenea, Gyrodactylidae). Evolution 56, 2445–2458. 12583585 10.1111/j.0014-3820.2002.tb00170.x

